# Brassinosteroids Inhibit Autotropic Root Straightening by Modifying Filamentous-Actin Organization and Dynamics

**DOI:** 10.3389/fpls.2020.00005

**Published:** 2020-02-04

**Authors:** Louise de Bang, Ana Paez-Garcia, Ashley E. Cannon, Sabrina Chin, Jaydeep Kolape, Fuqi Liao, J. Alan Sparks, Qingzhen Jiang, Elison B. Blancaflor

**Affiliations:** ^1^Noble Research Institute LLC, Ardmore, OK, United States; ^2^Department of Plant and Environmental Sciences, University of Copenhagen, Copenhagen, Denmark; ^3^Department of Biological Sciences, University of North Texas, Denton, TX, United States; ^4^Center for Biotechnology, University of Nebraska—Lincoln, Lincoln, NE, United States

**Keywords:** autotropism, actin, brassinosteroids, clinostat, cytoskeleton, gravitropism, root development

## Abstract

When positioned horizontally, roots grow down toward the direction of gravity. This phenomenon, called gravitropism, is influenced by most of the major plant hormones including brassinosteroids. Epi-brassinolide (eBL) was previously shown to enhance root gravitropism, a phenomenon similar to the response of roots exposed to the actin inhibitor, latrunculin B (LatB). This led us to hypothesize that eBL might enhance root gravitropism through its effects on filamentous-actin (F-actin). This hypothesis was tested by comparing gravitropic responses of maize (*Zea mays*) roots treated with eBL or LatB. LatB- and eBL-treated roots displayed similar enhanced downward growth compared with controls when vertical roots were oriented horizontally. Moreover, the effects of the two compounds on root growth directionality were more striking on a slowly-rotating two-dimensional clinostat. Both compounds inhibited autotropism, a process in which the root straightened after the initial gravistimulus was withdrawn by clinorotation. Although eBL reduced F-actin density in chemically-fixed *Z. mays* roots, the impact was not as strong as that of LatB. Modification of F-actin organization after treatment with both compounds was also observed in living roots of barrel medic (*Medicago truncatula*) seedlings expressing genetically encoded F-actin reporters. Like in fixed *Z. mays* roots, eBL effects on F-actin in living *M. truncatula* roots were modest compared with those of LatB. Furthermore, live cell imaging revealed a decrease in global F-actin dynamics in hypocotyls of etiolated *M. truncatula* seedlings treated with eBL compared to controls. Collectively, our data indicate that eBL-and LatB-induced enhancement of root gravitropism can be explained by inhibited autotropic root straightening, and that eBL affects this process, in part, by modifying F-actin organization and dynamics.

## Introduction

Plants are sessile organisms that are limited by their location. As such, they have evolved sensitive and delicate ways to respond to environmental cues allowing them to reprogram their growth when presented with changes in their surroundings. One of the adaptive behaviors that plants exhibit in response to external signals is the ability to redirect the growth of their major organs towards or away from a perceived stimulus. These phenomena, collectively called tropisms, shape the above and belowground architecture of the plant. One of the most widely studied tropisms is gravitropism, which describes the growth of shoots and roots away or toward the gravity vector, respectively. Gravity perception occurs in the columella cells located in the root cap or endodermal cells in shoots. Columella and endodermal cells contain starch-filled amyloplasts, called statoliths, which sediment to the lower side of the cell when the plant is reoriented relative to the gravity vector (Sack, 1997; Tasaka et al., 1999; Kiss, 2000). The physical signal triggered by statolith sedimentation is transduced to the growing regions of the organ resulting in differential growth between the upper and lower sides (Blancaflor and Masson, 2003; Sato et al., 2015).

Plant organs typically have a defined gravitropic set point angle (GSA). Although the GSA of a specific organ is influenced by the developmental stage of the plant and other environmental cues, it is under tight genetic control allowing for the maintenance of a defined growth angle relative to gravity when other external signals are kept constant. For instance, under carefully controlled laboratory conditions, the main shoot axis typically grows vertically upward (negative gravitropism) with a GSA of 180˚ and the primary root grows vertically downward (positive gravitropism) with a GSA of 0˚. Lateral roots and shoot branches typically have a GSA between these values (Firn and Digby, 1997).

Recently, mechanisms underlying GSA of plant organs that exhibit non-vertical growth, such as lateral roots and shoot branches, have been shown to be dependent on auxin and plant nutritional status (Roychoudhry et al., 2013; Roychoudhry et al., 2017). Most studies on gravitropism have focused on understanding biological processes that lead to the initial redirection of organ growth when the plant is reoriented. However, gravitropism has been viewed as a composite response that includes restrained or anti-gravity growth readjustment in which the organ eventually straightens after the initial growth redirection. The latter phenomenon is known as autotropic straightening or autotropism (Stankovic et al., 1998a; Stanković et al., 1998b). Although it is well established that the initial gravitropic response (i.e. downward root growth and upward shoot growth) is modulated by auxin redistribution (Swarup et al., 2001; Rakusova et al., 2015), there is accumulating evidence that most of the major plant hormones participate in gravitropism through overlapping signaling pathways (Guisinger and Kiss, 1999; Lofke et al., 2013; Vandenbussche et al., 2013; Pernisova et al., 2016). By contrast, little is known about the underlying mechanisms regulating autotropism. It has been debated whether induction of autotropic straightening is triggered by gravity itself (Myers et al., 1995) or as an autonomic counter response to gravitropic bending (Tarui and Iino, 1999; Bastien et al., 2013; Okamoto et al., 2015). It has also been suggested that the term autotropism should be used exclusively in situations when plant organs straighten after withdrawal of the gravity stimulus through randomization of the gravity vector on a clinostat or in microgravity (Stanković et al., 1998b).

The cytoskeleton has been proposed to play major roles in all phases of gravitropism (Blancaflor, 2002; Bisgrove, 2008). Microtubules and filamentous-actin (F-actin) are the core protein components of the cytoskeleton, and their dynamics and higher order organization within the cell are defined by many regulatory proteins (Blancaflor, 2013). Most studies implicating the cytoskeleton in gravitropism have employed pharmacological approaches in which roots or shoots were exposed to cytoskeletal inhibitors, and the subsequent response of the organ to a horizontal reorientation was quantified (Blancaflor, 2002). Interestingly, such approaches have revealed that chemical inhibitors of F-actin such as latrunculin B (LatB) or cytochalasin B can elicit stronger gravitropic responses in shoots and roots (Blancaflor and Hasenstein, 1997; Yamamoto and Kiss, 2002; Blancaflor et al., 2003; Hou et al., 2003; Hou et al., 2004). The stronger gravitropic response of roots and shoots after treatment with F-actin antagonists was recapitulated in *Arabidopsis thaliana* mutants disrupted in vegetative actin isoforms (Kato et al., 2010; Lanza et al., 2012). Furthermore, the *A. thaliana shoot gravitropic response9* (*sgr9*) mutant, which had reduced shoot gravitropism and statolith sedimentation, could be rescued by LatB treatment (Nakamura et al., 2011). Recently, delayed root gravitropism in *A. thaliana actin-related protein 3* (*arp3*) mutants was linked to abnormal amyloplast sedimentation and bundled F-actin in the columella cells. The delay in root gravitropism was reversed by LatB exposure (Zhou et al., 2016). It was postulated that actin disruption promoted gravitropism by increasing gravity sensing potential through unimpeded statolith sedimentation (Blancaflor, 2013). However, this hypothesis remains to be tested experimentally.

As noted, gravitropism is modulated by most of the major plant hormones, but in the context of past actin inhibitor studies, brassinosteroid is the plant hormone that is of most interest. Like LatB, eBL promoted gravitropism in shoots and roots (Meudt, 1987; Kim et al., 2000; Li et al., 2005; Kim et al., 2007; Lanza et al., 2012). The effect of eBL on root gravitropism was explained primarily through its interaction with auxin (Kim et al., 2000; Kim et al., 2007). In *A. thaliana*, eBL induced the accumulation of the auxin efflux carrier PIN-FORMED 2 (PIN2) protein in the root elongation zone (Li et al., 2005). The effect of eBL on PIN2 accumulation was proposed to involve the actin cytoskeleton through an increase in the expression domains of ROP2 (for Rho of Plants), a small GTPase regulator of F-actin dynamics (Li et al., 2005). Furthermore, it was shown that exogenous eBL treatment altered both actin organization and PIN2 polar localization in *A. thaliana* roots in a manner similar to that of auxin (Lanza et al., 2012). Taken together, the actin cytoskeleton appears to be a component of the signaling pathways by which eBL promotes gravitropic growth responses in plants.

The promotive effect of LatB and eBL on gravitropism has been explained primarily in regard to their impact on gravity sensing events or on the differential growth responses resulting from altered auxin redistribution (Hou et al., 2003; Hou et al., 2004; Li et al., 2005; Lanza et al., 2012; Blancaflor, 2013). However, a recent study with *A. thaliana* mutants disrupted in genes encoding the actin motor protein myosin presents an alternative hypothesis to explain the LatB- or eBL-induced enhancement of root gravitropism. The apparent promotion of tropistic organ responses brought about by altered actin function may instead be due to its inhibition of the autotropic straightening response (Okamoto et al., 2015). In the study by Okamoto et al. (2015), inflorescence stems of *A. thaliana* myosin XI mutants bent strongly when the initial gravity stimulus was withdrawn by clinorotation, an observation that mirrored the persistent curvature of *Z. mays* roots treated with LatB (Hou et al., 2003). Here, we show that eBL-treated roots exhibited persistent curvature responses when the initial gravity response was withdrawn by clinorotation in a similar manner as LatB-treated roots. Our results indicate that the apparent promotion of root gravitropism by eBL can be explained by its inhibitory effect on autotropic straightening that resulted, in part, from eBL-mediated alterations in F-actin dynamics and organization.

## Materials and Methods

### *Zea mays* Root Growth and Drug Treatments

Maize (*Zea mays* L. cultivar Merit) seeds were soaked for 10 to 12 h in deionized water. After soaking, seeds were planted side by side with the embryo facing down on 45 × 35 cm opaque plastic trays lined with moist germination paper (Seedburo Equipment Co. USA). The seeds were covered with a second layer of germination paper and gentle pressure was applied to the paper so that surface tension between the two sheets of paper held the seeds in place. A second opaque plastic tray was placed on top of the first tray so that the two layers of germination paper containing the seeds were secured between the two trays. The trays were oriented vertically in a plastic tub filled with water to a depth of 5–10 cm to keep the germination paper moist. After 48 h, seedlings with 1–2 cm long straight primary roots were selected and treated with working solutions of LatB, eBL or the corresponding solvent control, which consisted of diluted dimethyl sulfoxide (DMSO). To treat roots, a set-up consisting of 1.5 ml microfuge tubes oriented vertically on microfuge holders was prepared. The center of the lid of the microfuge tube was punctured with the tip of a heated metal probe to create a 2 mm diameter hole, and 1 ml of the treatment solution was added to each tube. Selected seedlings were transferred one at a time to each microfuge tube by inserting the roots into the hole so that 20 mm of the root tip was immersed vertically in the solution. Seedlings were stabilized during treatment by mounting the kernel to the microfuge tube with caulking compound. Roots were incubated with 100, 250, 500, and 1,000 nM LatB and eBL and DMSO controls using this set-up for 1 h. Soaking, germination and treatment was done in a Conviron growth chamber (Controlled Environments Ltd, Canada) set to 24°C with 14 h (120 μmoles m^-2^ s^-1^)/10 h day/night cycle.

Stock solutions of LatB (1mM; Calbiochem, USA) and eBL (10 mM; Santa Cruz Biotechnology, USA) were prepared with 100% (v/v) DMSO (Sigma-Aldrich, USA) and stored at -20°C prior to use. Working solutions were prepared by adding the appropriate volume of stock solution to sterile de-ionized water.

### *Z. mays* Clinorotation Assays

After treatment, five seedlings were selected from each treatment group and mounted on 10 × 10 cm square Petri dishes with double-sided foam tape. The lid of the Petri dish was lined with two layers of wet germination paper (Seedburo Equipment Co., USA) to maintain high humidity. The Petri dishes were sealed with parafilm and mounted on a custom-built two-dimensional (2-D) clinostat located in the same Conviron growth chamber (Controlled Environments Ltd, Canada) used for seed germination and root treatment. The Petri dishes were first mounted so that the roots were vertical (**Appendix 1**). After 60 min the Petri dishes were rotated by 90° so that roots were in a horizontal position. After 20 min, the clinostat was activated and roots were rotated for 20 h at 1 revolution per minute (RPM). Digital images of the roots after clinorotation were captured using a camera mounted on a copy stand. Each treatment was replicated three times using a total of 15–20 seedlings. Root curvature was measured using ImageJ software (https://imagej.nih.gov/ij/).

### Time-Lapse Imaging of Graviresponding *Z. mays* Roots

Another set of *Z. mays* seedlings was treated with 100 nM LatB, 500 nM eBL and the solvent control solution and mounted on square Petri dishes following the methods described above. The plates were positioned so that the roots were in a vertical orientation. After 60 min, the plates were rotated 90° so that the roots were horizontal. Immediately after rotation, images were captured every 15 min for 12 h. Time-lapse movies were generated using the Metamorph 5.0 image acquisition software (Universal Imaging Corp, USA). From the generated time-lapse movies, the root tip angle with respect to the horizontal was measured using ImageJ software. Experiments were repeated at least three independent times with 15-20 seedlings per treatment.

### F-Actin Labeling and Confocal Microscopy of Fixed *Z. mays* Roots

Labeling and imaging of F-actin was essentially as described by Dyachok et al. (2016). Briefly, the apical 5 mm of roots incubated in LatB, eBL and the corresponding solvent control for 1 h were fixed in 50 mM piperazine- N,N ′ bis [2-ethansesulfonic acid], 2 mM MgCl_2_, and 10 mM ethylene glycol-bis(β-aminoethyl ether)-N,N,N′,N′-tetraacetic acid pH 7.0 (PME buffer) containing 5% DMSO and 2% paraformaldehyde (Electron Microscopy Sciences, USA). After roots were washed with PME buffer, they were sectioned at a thickness of 70 μm using a Vibratome 1500 (Ted Pella Inc., USA). F-actin in the root sections was labelled with Alexa Fluor 488-phalloidin (Molecular Probes, USA) suspended in PME buffer. After mounting sections on glass slides, they were imaged with an inverted Leica TCS-SP8-X confocal laser scanning microscope (Leica Microsystems, Germany) equipped with a 63× oil-immersion objective. Alexa-Fluor was excited with the 488 nm line of the white light laser and emission was detected at 522 nm. To enhance the resolution of F-actin images obtained from fixed roots for downstream quantitative analysis, the HyVolution deconvolution package (Huygens, The Netherlands) was used to process image data directly acquired with the Leica SP8-X system. Five optical sections taken at 0.5 μm intervals were acquired. Each dataset was then processed using the wizard-driven HyVolution user interface linked with the Leica Application Suite (LAS) software.

### Generation of *M. truncatula* Plants Expressing Fluorescent Protein-Based F-Actin Reporters

Transgenic *M. truncatula* lines expressing *UBQ10:Lifeact-mGFP* and *UBQ10:GFP-ABD2-GFP* were generated essentially as described in Jiang et al. (2019). Briefly, *Agrobacterium tumefaciens* strain EHA 105 harboring Lifeact and ABD2 constructs were streaked on Luria-Bertani (LB) agar plates containing 50 mg ml^-1^ kanamycin and 25 mg ml^-1^ rifampicin and cultured at 28°C for 2–3 days. A single colony was picked and then cultured in LB liquid media containing the same antibiotics for 16 h or until the OD600 was 0.6–0.8. The trifoliates located second from the top were collected from 4–6 week-old *M. truncatula* R108 plants grown at 24/20°C and 16/8 h day/night. The leaves were sterilized with 20% commercial bleach containing a drop of Tween-20 for 10 min and then washed three times with sterile water. The leaves were then infected with the *A. tumefaciens* suspension that was generated by centrifuging the *A. tumefaciens* liquid culture at 3,500 rpm for 20 min and then resuspending the pellets in liquid infection medium to an OD600 of 0.2–0.3. The infection procedure included a 10-min vacuum infiltration, a 3–5 min sonication and then another 10-min vacuum infiltration. After this, the infected leaves were blot dried and plated on co-cultivation medium. The trifoliate explants were transferred onto selection medium containing 10 mg ml^-1^ hygromycin after 24–30 h incubation under 24°C in the dark. Culture of the explants continued for an additional 5–6 weeks under the same conditions or until enough resistant calli were produced. The resistant calli were then transferred onto shoot regeneration medium and cultured under 150 μmoles m^-2^ s^-1^ light at 24/20°C and 16/8 h day/night cycle. After 2–3 months, plantlets were fully regenerated with functional roots. A couple of leaflets were sampled from the regenerated plants for DNA extraction. Genomic DNA was extracted using cetyl trimethylammonium bromide. DNA samples were PCR amplified with primers for *hygromycin B phosphotransferase* gene (forward 5’- AAGGAATCGGTCAATACACTACATGG -3’ and reverse 5’- AAGACCAATGCGGAGCATATACG -3’). Confirmed transgenic plants were then screened using a fluorescent stereo microscope and those with GFP signal were transferred to soil and allowed to go to seed. Multiple, independent lines of both *UBQ10:GFP-ABD2-GFP*, and *UBQ10:Lifeact-mGFP* were screened in the subsequent generations until homozygous positive lines were obtained.

### Growth Characterization of *M. truncatula* F-Actin Reporter Lines

Seeds of both reporter lines and wild type (R108) were scarified in concentrated sulfuric acid for 8 min, washed with cold water, and then surface sterilized for 3 min in a solution containing 30% commercial bleach and 0.1% Tween-20. After decanting the bleach solution, the seeds were rinsed and stored overnight in the dark at room temperature. The seeds were placed onto sterile, moist filter paper in Petri dishes and stratified at 4°C in the dark for 2 days and then transferred to a growth chamber at 22°C and 16/8 h day/night (modified from Boisson-Dernier et al. (2001). After germinating, seedlings were transferred to square agar plates supplemented with ½ Murashige and Skoog medium. The plates were moved back to the growth chamber and positioned vertically to allow the roots to elongate on the agar media surface. The root tip of at least 16 plants per genotype was marked with a black dot. This process was repeated two times every 24 h for a total of 3 days. ImageJ was used to measure the average root elongation per day and this measurement was used as an indicator of the root growth rate.

Plants were allowed to grow for four more days in plates, for a total of 7 days. The plates were imaged and RootNav software (University of Nottingham, UK) was used to automatically measure the primary root length (Pound et al., 2013). The plants were then transferred to pots filled with a mixture of turface and vermiculite (3:1), and then grown at 22°C and 16/8 h day/night for three more weeks, for a total of 28 days of growth. After this time, the plants were removed from the pots and the soil mixture was washed away from the roots. Roots and shoots were separated, bagged, and dried at 65°C for one week, before obtaining shoot and root dry weight.

### Clinorotation and F-Actin Imaging of *M. truncatula* Expressing F-Actin Reporters

*M. truncatula* seeds were scarified with 150 grit sandpaper (3M, USA). Next, seeds were sterilized with 6% (v/v) sodium hypocholorite in 50 ml Falcon tubes (Corning, USA) for 10 min in room temperature, with intermittent rotation. Post-incubation, the seeds were rinsed six times with sterile deionized water and were spread out on 10% (w/v) agar (Sigma- Aldrich, USA) plates in 100 × 25 mm round petri dishes. Seeds were stratified for 2 days in the dark at 4°C. Plates were flipped upside-down and seeds were germinated by transferring them to a 24°C incubator for 20 h. Six seedlings with straight radicles that emerged into the air were selected and transferred onto a 10 ×10 cm square plates containing 10% (w/v) agar (Sigma-Aldrich, USA) for subsequent treatment with germination paper (Seedburo Equipment Co., USA) that was pre-soaked with either 100 nM LatB, 5 μM eBL or DMSO solvent control. The soaked germination paper was laid on the radicles of the seedlings for an hour in room temperature and the plate was set up vertically to enable radicles to continue growing vertically straight.

Plates for clinorotation assays were prepared by filling 10 × 10 cm square plates with 10% (w/v) agar (Sigma-Aldrich, USA) up to half of the height of the plate. Once the agar had set, half of the agar was cut and removed from the plate with a razor blade. In addition, six shallow indentations for the cotyledons of six seedlings was cut with a razor blade. Each seedling was fitted into its respective indentation, with its cotyledon held by the agar and its radicle suspended into the air in the plate. Next, 52°C molten agar was pipetted on the cotyledons to hold each seedling firmly in its location. After growing seedlings vertically for 1 h, the plates were rotated 90° for 20 min followed by clinorotation for 15 h at 1 rpm on the clinostat. Post-treatment, images of the roots were taken using a camera mounted on a copy stand and root curvature was measured using ImageJ software.

To image F-actin, 2-day-old seedlings with straight roots were transplanted onto 64 × 48 mm cover slips coated with a 2 mm thick slab of 1% agar. Transplanting was done by gently lifting the agar slab and placing the root on top of the cover slip. The lifted agar was then used to hold the root in place so that it lay between the agar and coverslip. Roots treated with LatB and eBL for 1 h were imaged immediately. Another set of transplanted seedlings was used to evaluate the quality of F-actin labeling in non-treated seedlings. For this set of seedlings, the cover slip with transplanted seedlings was placed in a 9 cm round Petri dish and transferred to the same Conviron (Controlled Environments Ltd, Canada) used for *Z. mays* clinostat assays. The Petri dishes were tilted at an angle of 60° to enable the root to grow close to the bottom surface of the coverslip. One to 2 days after transplanting, seedling roots were imaged with a Leica SPX-8 confocal microscope (Leica Microsystems, Germany).

### Live Cell Imaging of Dark Grown *UBQ10:Lifeact-mGFP M. truncatula* Hypocotyls

*M. truncatula* seeds expressing *UBQ10:Lifeact-mGFP* were stratified on agar plates with ½ Murashige and Skoog medium at 4°C for 12–24 h. Seeds were exposed to light for 4 h and then kept in darkness for 3–5 days in a Conviron (Controlled Environments Ltd, Canada) set to 24°C and 14 h (120 μmoles m^-2^ s^-1^) and 10 h day/night cycle. The elongated hypocotyls were incubated in 500nM eBL and DMSO solvent control solution for 1 h. Fine actin filaments of epidermal cells adjacent to the hook region were imaged every second for 1 min with an UltraView ERS spinning-disk confocal microscope (Perkin-Elmer, USA) equipped with a 100× oil immersion objective (Staiger et al., 2009).

### Quantification of F-Actin Organization and Dynamics of *Z. mays* Fixed Roots and *M. truncatula* Live Roots

For occupancy, skewing and eccentricity analysis, HyVolution processed images from fixed maize roots and live barrel medic roots were cropped manually using Adobe Photoshop CS5 so that only individual root cells occupied the image field of view. Each of the cropped images was processed by canceling the background noise followed by marking F-actin with one pixel-wide lines following the methods described in (Wang et al., 2008; Vidali et al., 2009; Dyachok et al., 2014; Sparks et al., 2016). Eccentricity was calculated from compact ellipse sections from cropped root images. This process included extraction of the intensity distribution of a low frequency spectrum at 95% CI (Confidence Interval) after applying fast Fourier transform (FFT) to the F-actin images following Dyachok et al. (2011); Nakashima et al. (2014) and Burkart et al. (2015). Therefore, eccentricity reflects the alignment properties of F-actin within each cell. Algorithms and software for occupancy and eccentricity analysis were written in MATLAB and used to automatically analyze all cell samples.

Quantitative analysis of cell dynamics from movies obtained from an UltraView ERS spinning-disk confocal microscope (Perkin-Elmer, USA), was conducted essentially as described in Dyachok et al. (2014) and Vidali et al. (2010). The algorithms and software were developed in MATLAB. Briefly, the software extracted 60 frames in the movie (1 frame per second). The software calculated the correlation and difference of each frame by averaging correlation and difference between this frame and all other frames. The correlation was defined as two-dimensional correlations between two matrixes, and the difference was considered by subtracting two matrixes after preprocessing for each matrix.

## Results

### Epi-Brassinolide-Treated *Z. mays* Roots Exhibit Persistent Curvature on a Slowly Rotating Clinostat and Enhanced Gravitropism

**Figure 1:**
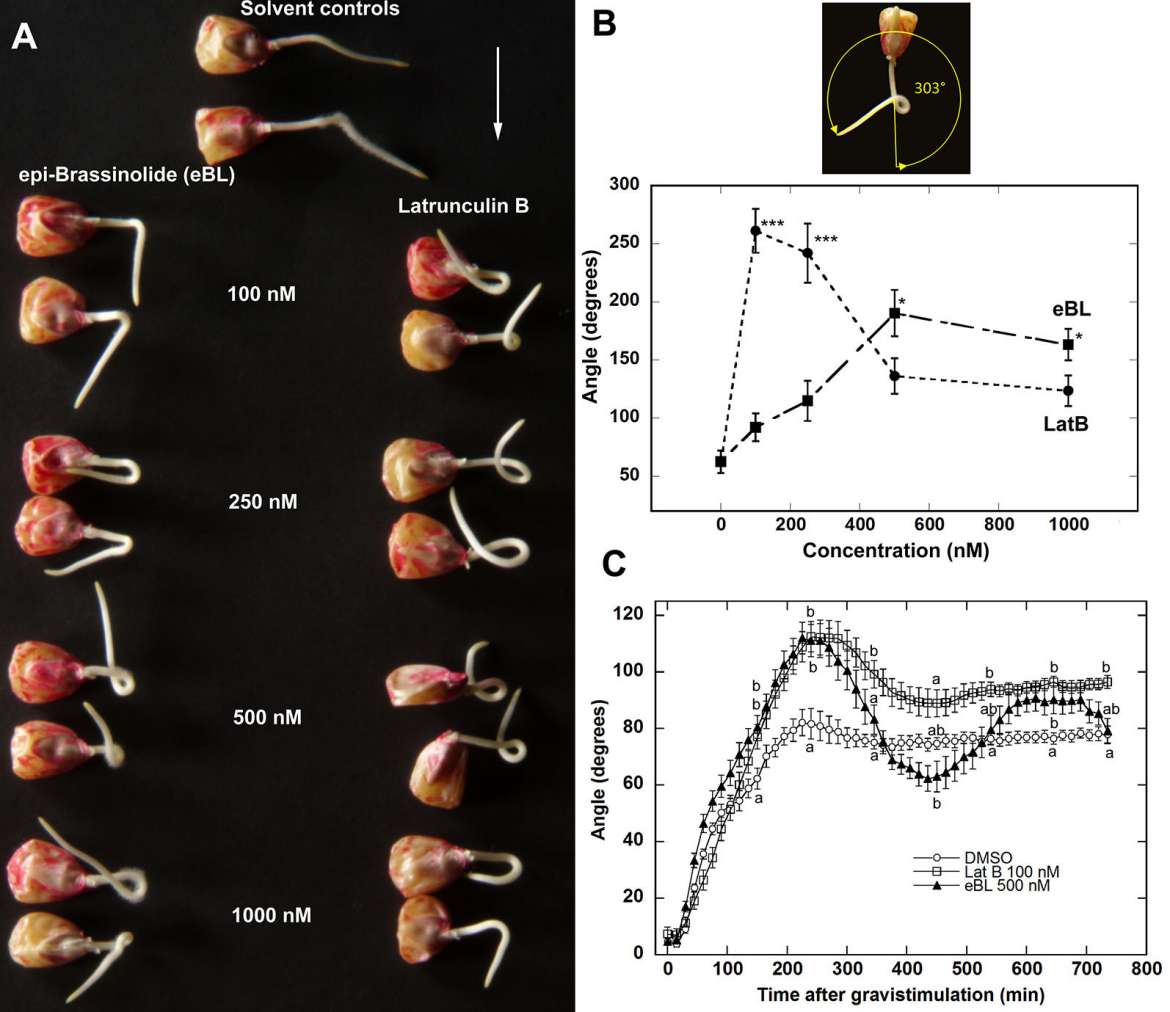
Epi-Brassinolide (eBL) and LatB enhanced gravitropism and dampened straightening of *Z. mays* roots. **(A)** Representative images of *Z. mays* roots grown on a 2-D clinostat for 20 h to test autotropic straightening after withdrawal of the gravity signal. Vertical roots treated with LatB and eBL for 1 h were mounted on a 2-D clinostat and kept horizontal for 20 min (white arrow indicates the direction of gravity) prior to clinorotation for 20 h. Note that untreated roots (solvent controls) straightened after reaching an angle of about 45° while roots treated with eBL or LatB did not. **(B)** Dose response of root curvature on a clinostat after eBL and LatB treatment. Angle of curvature was obtained as shown by the yellow arrow above the graph. Bars are means (n > 14 plants) ± S.E. ***P < 0.001, * P < 0.05 (Student’s t-test). **(C)** Time course of root gravitropism after vertically growing roots were placed horizontally without clinorotation. Statistical significance was determined by one-way ANOVA. Means (n > 12 plants) ± S.E. Different letters indicate significant differences among means (P < 0.05, Tukey’s test).

Previous reports have shown that LatB and eBL promoted gravitropism in roots (Hou et al., 2003; Hou et al., 2004; Li et al., 2005; Kim et al., 2007; Lanza et al., 2012). The enhancement of gravitropic response in LatB-treated roots was more strongly manifested on a slowly-rotating 2-D clinostat. For instance, *Z. mays* roots treated with LatB displayed persistent root curvature on the clinostat, as opposed to untreated roots that straightened (Hou et al., 2003). We hypothesized that the enhanced gravitropism of eBL-treated roots was likely to also be more prominent under clinorotation. Our experiment involved applying a perpendicular gravistimulus to roots for 20 min after pre-treating them for an hour with LatB or eBL followed by clinorotation for 20 h (**Appendix 1**). We found that eBL-treated roots mirrored the persistent curvature responses of LatB-treated roots (**Figure 1A**; Hou et al., 2003). Quantification of the angle of root curvature that developed on the clinostat revealed that LatB was more potent than eBL when tested at the same concentrations. For instance, LatB induced a strong curvature response at 100 nM concentration, whereas eBL elicited a similar curvature at a higher 500 nM concentration (**Figure 1B**).

Next, we compared the kinetics of gravitropism of *Z. mays* roots pretreated for 1 h with 100 nM LatB or 500 nM eBL under continuous gravistimulation (i.e. roots reoriented 90° from the vertical without clinorotation). We found that roots treated with the solvent control solution grew downward and reached a maximum angle of about 80° from the horizontal at approximately 200 min. Control roots maintained this angle for the entire 720 min time course. By contrast, LatB- and eBL-treated roots overshot the vertical and reached maximum angle of approximately 110° after 250 min (**Figure 1C**). Overshooting of the vertical by LatB- and eBL- treated roots was followed by a correction to the vertical that began at about 300 min after orienting roots horizontally. Although both LatB- and eBL-treated roots exhibited similar kinetics with regard to their overshooting of the vertical, the subsequent overcorrection was more pronounced in eBL-treated roots. eBL-treated roots returned to an angle of about 60° at 450 min after gravistimulation, but eventually stabilized to a 90° growth angle by about 600 min. At the end of the 720 min time course, eBL-treated roots reached a similar angle as controls. By contrast, LatB-treated roots returned to about 90° at 450 min and maintained this growth angle for the duration of the time course (**Figure 1C**).

### Epi-Brassinolide Modifies F-Actin Organization in *Z. mays* Roots

**Figure 2:**
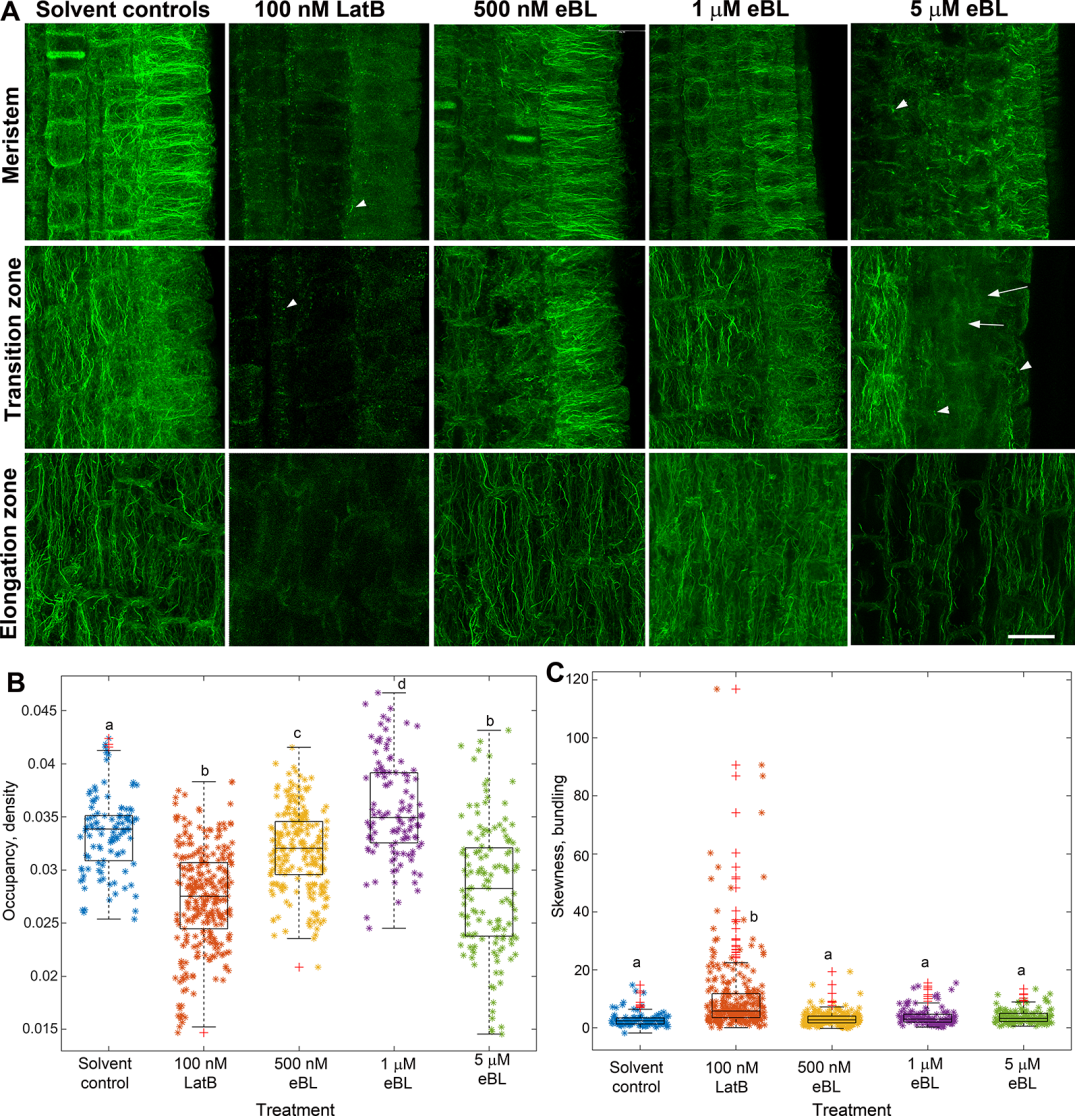
Epi-Brassinolide (eBL) modified F-actin organization in *Z. mays* roots. **(A)** Single optical confocal micrographs of chemically-fixed and longitudinally-sectioned *Z. mays* roots stained with Alexa Fluor 488-phalloidin. Note that LatB-treated roots had diffuse staining and fragmentation of F-actin in the form of puncta and short filaments (arrowheads). Untreated (solvent controls) had extensive F-actin networks. 500 nM and 1 µM eBL on the other hand did not result in any visible effects on F-actin. At 5 µM eBL, some regions of the meristem, transition and elongation zone have diffuse fluorescence (arrows) and fragmented F-actin (arrowheads). **(B**, **C)** Combined box and scatter plots showing quantitative analysis of F-actin occupancy (density **B**) and skewness (bundling **C**) in root cells of the transition/elongation zone. Statistical significance was determined by one-way ANOVA. Asterisks (*) refers to individual data points and + mark outliers. Bars are means (n > 100 cells) ± S.E. Different letters indicate significant differences among means (P < 0.05, Tukey’s test). Scale bars = 20 μm.

The similar effects of LatB and eBL on maize root gravitropism and autotropic root straightening on a 2-D clinostat (see **Figure 1**) led us to hypothesize that eBL may cause LatB-like effects on the actin cytoskeleton. To test this hypothesis, we studied F-actin organization in maize roots that were treated with LatB and eBL at concentrations that produced comparable root phenotypes (i.e.100 nM for LatB and 500 nM for eBL). After exposure to the compounds, roots were chemically fixed, sectioned and F-actin was labeled with phalloidin conjugated to a fluorophore (Dyachok et al., 2016). Consistent with our previous studies (Hou et al., 2003), distinct qualitative differences in F-actin organization were observed between roots treated with solvent controls and roots treated with 100 nM LatB in the meristem, transition zone and elongation zone. Whereas controls had extensive F-actin networks in various root developmental zones, LatB-treated roots had diffuse labeling or severely fragmented F-actin manifested primarily as bright puncta (**Figure 2A**). By contrast, roots treated with 500 nM eBL still had abundant F-actin networks in the various root developmental zones that appeared similar to control roots. Since roots treated with 500 nM eBL did not show obvious qualitative differences in F-actin organization compared with control roots, we increased the eBL treatment concentration to 1 and 5 µM. Nonetheless, even after treatment with 1 µM eBL, F-actin in various regions of the root appeared similar to untreated roots. Qualitative disruptions to F-actin only became obvious when eBL concentration was increased to 5 µM. At this concentration some root cells had diffuse fluorescence and fragmented F-actin that resembled LatB-treatment to some degree (**Figure 2A**). However, unlike LatB-treated roots, roots treated with 5 µM eBL contained more cells with intact F-actin networks that were similar to untreated roots. This result suggested that while eBL disrupts actin, its effects were milder than LatB.

To quantify the effect of LatB and eBL on the root F-actin networks, we acquired occupancy values from images of root cells within the transition and elongation zone. We focused on these root zones because they have been shown to contribute most to the differential growth during the gravity response (Baluska et al., 1996). Occupancy is a quantitative metric used to depict F-actin density with higher values indicating a denser F-actin network (Higaki et al., 2010b). Compared with controls, occupancy of F-actin in roots treated with 100 nM LatB, 500 nM eBL, and 5 µM eBL was lower. Surprisingly, F-actin occupancy was significantly higher in roots treated with 1 µM eBL compared to control roots (**Figure 2B**). Taken together, our results show that eBL modifies F-actin organization in maize roots by causing a significant reduction in density. However, unlike roots treated with LatB, which showed more widespread disruption of F-actin, roots treated with eBL required higher concentrations (i.e. 5 µM) to detect distinct qualitative and quantitative effects on F-actin structure. Even at this higher concentration, impacts on F-actin appeared to be localized to random regions of the root (**Figure 2A**).

Another metric that we used to quantify eBL effects on F-actin structure is skewness, which measures the degree of asymmetry of fluorescence intensity distribution. Skewness has been shown to be a good indicator of F-actin bundling (Higaki et al., 2010a). We found that while LatB treatment resulted in a small but significant increase in bundling, eBL at all concentrations, did not (**Figure 2C**).

### Lifeact-mGFP and GFP-ABD2-GFP Reporters Decorate Extensive F-Actin Networks in Living *M. truncatula* Seedlings

**Figure 3:**
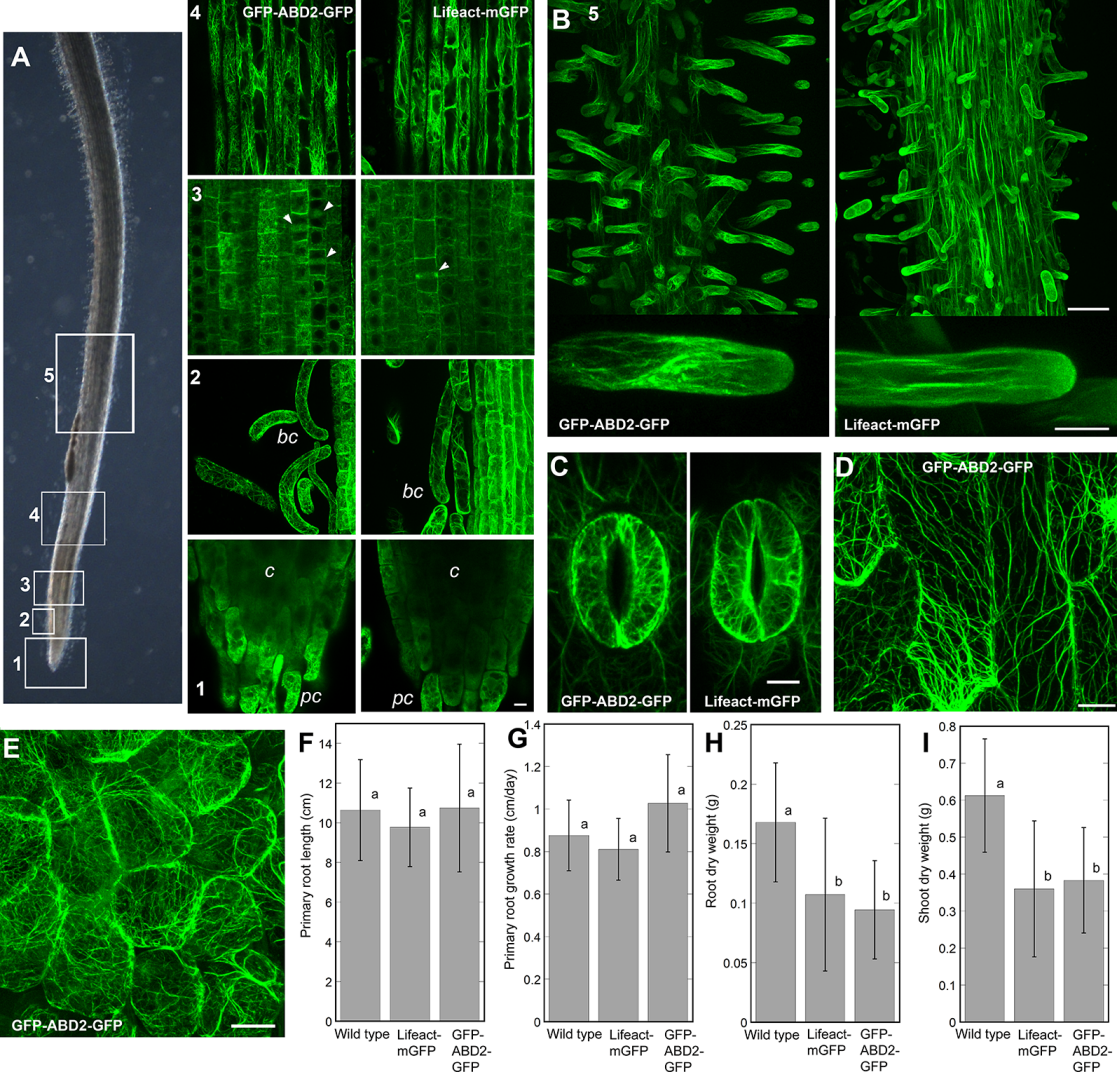
Representative images of F-actin in various cells and tissues of *M. truncatula* seedlings expressing the *UBQ10:GFP-ABD2-GFP and UBQ10:Lifeact-mGFP* reporters. **(A)** F-actin in various developmental regions of seedling primary roots. Numbers (1-5) in **(A**, **B)** correspond to regions of the bright field image of the root shown in (panel **A**). 1 = root cap, 2 = border cells, 3 = meristem 4 = elongation zone, 5 = root hairs. Note that while peripheral cap (*pc*) and border cells (*bc*) have distinct F-actin arrays, the columella (*c*) region has diffuse fluorescent signal. Arrowheads in the root meristem mark the phragmoplasts. **(B)** Both *UBQ10:GFP-ABD2-GFP and UBQ10:Lifeact-mGFP* decorate F-actin in growing root hairs. Extensive F-actin networks were also documented in guard cells of cotyledons **(C)**, epidermal cells of minor veins of a young leaf **(D)** and epidermal cells in a young nodule **(E)**. Scale bars = 10 µm (for **A**, **C**, **D**) 20 µm (for **E**), 50 µm (for low magnification images in **B**) and 10 µm (for high magnification images in **B**). Primary root length and primary root growth rate were not affected in *UBQ10:GFP-ABD2-GFP* and *UBQ10:Lifeact-mGFP* lines, compared to wild type **(F**, **G)**. Actin reporter lines showed smaller root and shoot dry weight when compared to wild type, after 28 days of growth **(H**, **I)**. Statistical significance was determined by one-way ANOVA. Bars are means (n > 16 plants) ± S.E. Different letters indicate significant differences among means (P < 0.05, Tukey’s test).

Our studies on chemically-fixed roots of *Z. mays*, support the hypothesis that the effects of eBL on root gravitropism and autotropic straightening on a 2-D clinostat are due to disruptions in normal actin function (**Figures 1** and **2**). However, the results indicate that eBL only had modest effects on overall F-actin organization based on qualitative and quantitative analyses of chemically-fixed *Z. mays* roots (**Figures 2A–C**). Therefore, we next asked if effects of eBL on F-actin would be more obvious in living roots. To address this question, transgenic *M. truncatula* plants that expressed two live cell F-actin reporters (*Lifeact-mGFP* and *GFP-ABD2-GFP*) under the control of the *ubiquitin 10* (*UBQ10*) promoter were generated (Wang et al., 2008; Vidali et al., 2009; Dyachok et al., 2014; Sparks et al., 2016). Before using the lines for clinorotation assays, we first examined the quality of F-actin labeling in seedling roots. We found that Lifeact-mGFP and GFP-ABD2-GFP decorated extensive F-actin networks in various developmental regions of seedling roots. Distinct F-actin arrays were present in border cells, meristematic cells and cells in the transition/elongation zone. Similar to previous reports in *A. thaliana*, both Lifeact-mGFP and GFP-ABD2-GFP reporters labeled end walls and phragmoplasts in the meristem, and random and longitudinal F-actin cables in the elongation zone of *M. truncatula* seedling roots (**Figure 3A**). In agreement with previously published work with fixed and living roots, both reporters did not label distinct F-actin networks in the columella despite the presence of clear F-actin bundles in the peripheral cap and border cells. Only diffuse fluorescence was observed in the central columella of both reporter lines (**Figure 3A**; Baluska et al., 1997; Blancaflor, 2013). Lifeact-mGFP and GFP-ABD2-GFP also labeled extensive F-actin arrays in growing root hairs that were reminiscent of F-actin organization in *A. thaliana* lines expressing these two reporters (**Figure 3B**; Dyachok et al., 2014; Sparks et al., 2016). Moreover, other cell types in transgenic lines expressing GFP-ABD2-GFP and Lifeact-mGFP including guard cells, minor leaf veins and epidermal cells in nodules exhibited dense F-actin arrays (**Figures 3C–E**). Although primary root length and growth rate of 5-day-old *M. truncatula* seedlings expressing *Lifeact-mGFP* and *GFP-ABD2-GFP* were similar to wild type (**Figures 3F, G**), dry weights of the same lines at 28 days were lower compared to wild type (**Figures 3H, I**).

### Epi-Brassinolide Triggers Enhanced Root Curvature Responses in *M. truncatula on* a Clinostat, But Only Had Mild Effects on F-Actin Organization

**Figure 4:**
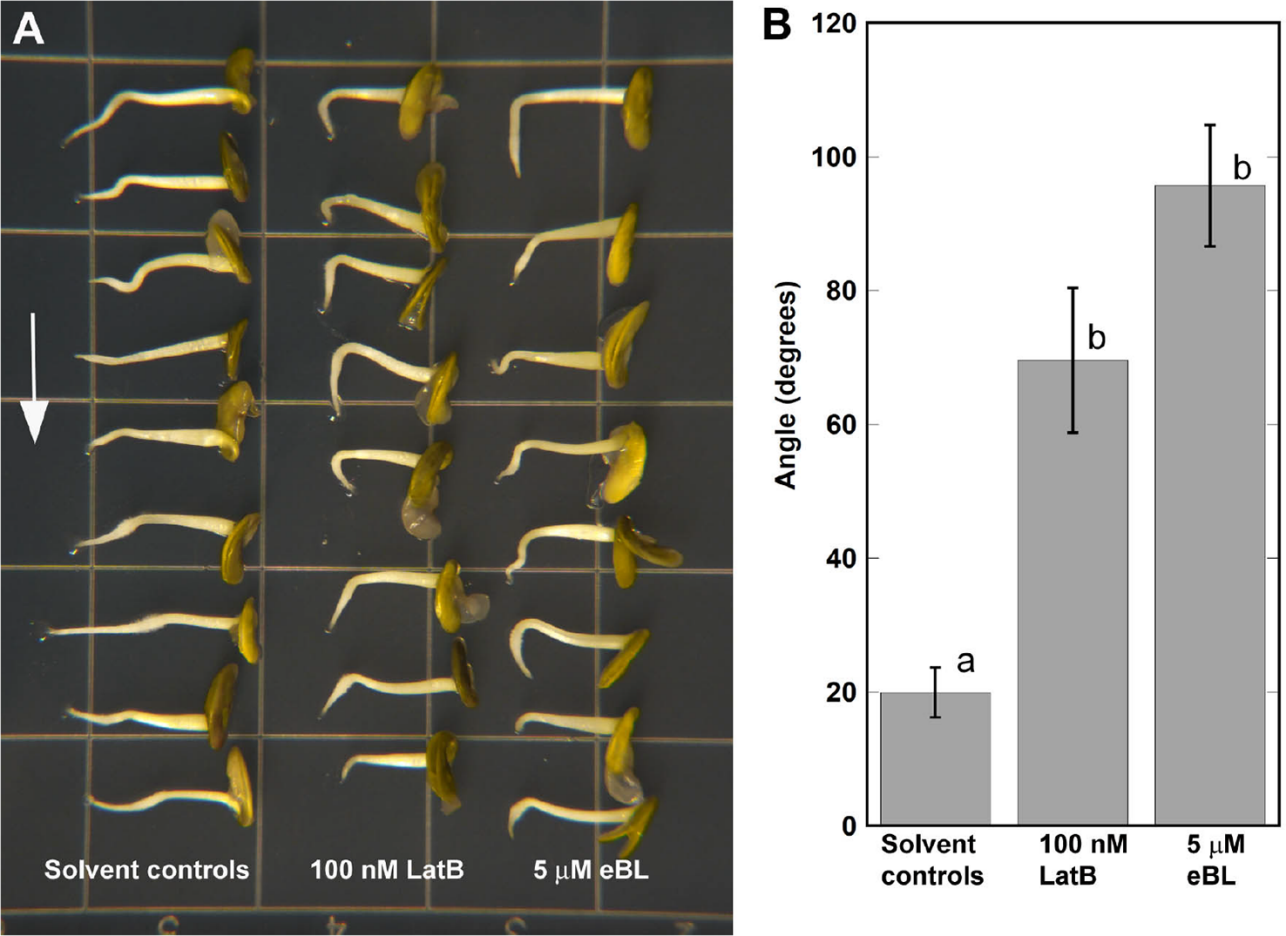
Epi-Brassinolide (eBL)- and LatB- dampened straightening of *M. truncatula* roots expressing *UBQ10:GFP-ABD2-GFP*. **(A)** Representative images of *M. truncatula* roots grown on a 2-D clinostat for 15 h to test autotropic straightening after withdrawal of the gravity signal. Vertical roots treated with 100 nM LatB and 5 μM eBL for 1 h were mounted on a 2-D clinostat and kept horizontal for 20 min prior to clinorotation. **(B)** Quantification of angle of curvature after 15 h of clinorotation. Statistical significance was determined by one-way ANOVA. Means (n > 14 roots) ± S.E. Different letters indicate significant differences among means (P < 0.05, Tukey’s test).

We next asked if eBL could elicit similar enhancement of root curvature responses in *M. truncatula* on a clinostat that was previously observed in *Z. mays* roots (**Figure 1**). As 5 μM eBL was required to observe clear effects on F-actin in chemically-fixed *Z. mays* roots (**Figure 2**), we used the same concentration for the *M. truncatula* clinostat assays. Furthermore, we focused our clinostat assays on lines expressing GFP-ABD2-GFP. The GFP-ABD2-GFP lines were chosen for our root clinostat assays because F-actin in the transition and elongation zone was finer and less bundled compared to Lifeact-mGFP lines, an observation consistent with previous work on *A. thaliana* (Dyachok et al., 2014). Consistent with previous reports, LatB-treated *M. truncatula* roots expressing GFP-ABD2-GFP exhibited enhanced curvature on a clinostat (**Figures 4A, B**; Hou et al., 2003). Like in *Z. mays* roots, eBL treatment caused *M. truncatula* roots to curve strongly on a clinostat (**Figures 4A, B**). Although, enhanced curvature on a clinostat was observed in LatB- and eBL-treated *M. truncatula* roots, the final average curvature was less than that observed in *Z. mays* (Compare **Figure 1** and **Figure 4**).

**Figure 5:**
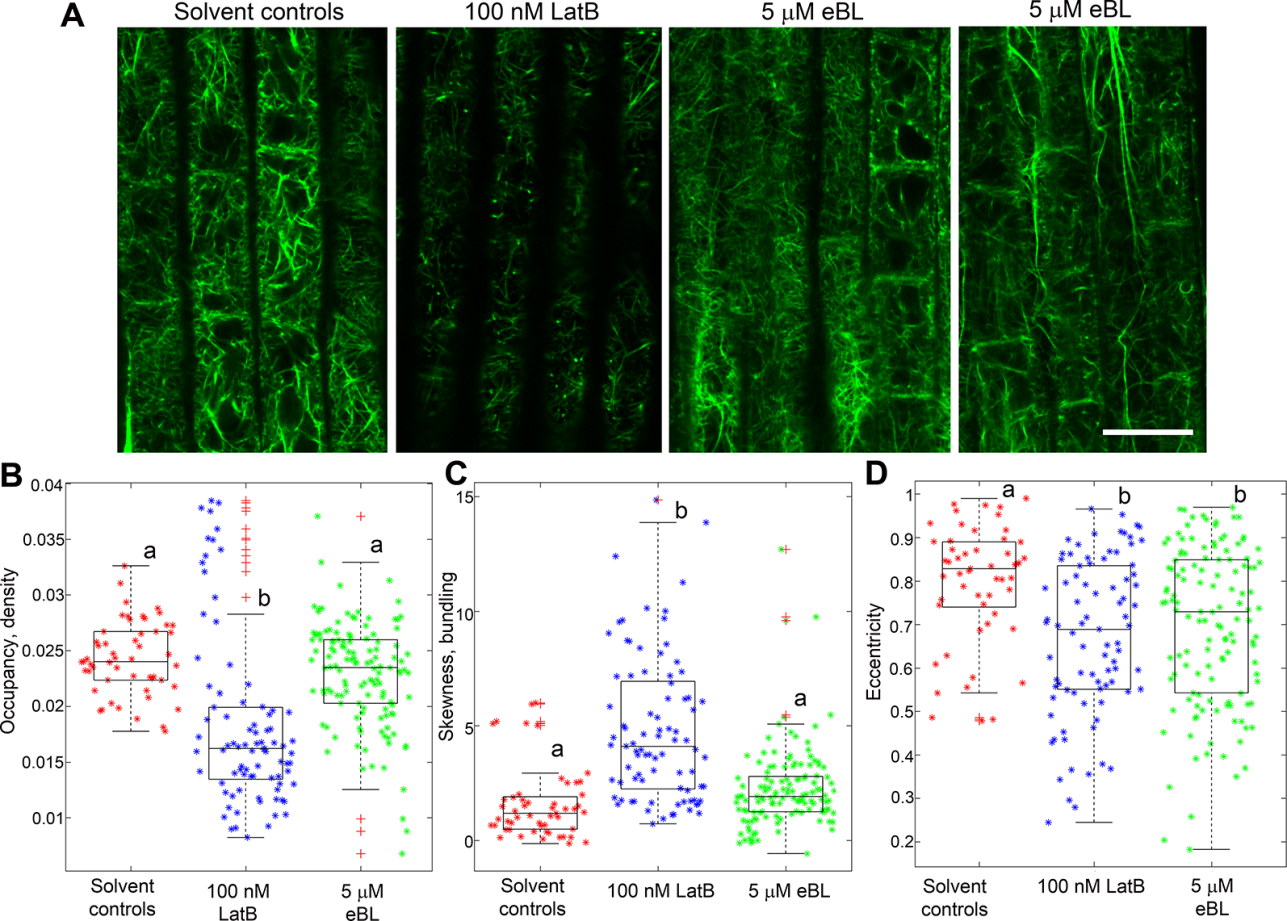
F-actin organization in epidermal cells of living *M. truncatula* roots expressing *UBQ10:GFP-ABD2-GFP* after 1 h exposure to LatB or eBL. **(A)** Representative single confocal optical section images of cells in the elongation zone. Note the reduced fluorescence and extensive fragmentation of F-actin in cells treated with LatB but not with eBL. The two right most panels show variable effects of eBL on F-actin organization. **(B**–**D)** Combined box and scatter plots showing quantitative analysis of F-actin occupancy (density), skewness (bundling) and eccentricity in root cells of the transition/elongation zone. Unlike LatB-treatment, no significant differences between eBL and solvent controls were detected with regard to F-actin occupancy **(B)** and skewness **(C)**. However, a significant difference was observed between eBL and solvent controls with regard to the eccentricity metric, which refers to alignment of F-actin **(D)**. Asterisks (*) refers to individual data points and + mark outliers. Statistical significance was determined by one-way ANOVA. Bars are means (n > 50 cells) ± S.E. Different letters indicate significant differences among means (P < 0.05, Tukey’s test). Scale bar = 20 μm.

Having established that *M. truncatula* roots expressing the GFP-ABD2-GFP F-actin reporter exhibited enhanced curvature on a clinostat, we then asked if F-actin organization in living roots was modified by eBL. We focused our analyses on root cells within the transition and elongation zone. Consistent with observations in chemically-fixed *Z. mays* roots, F-actin in *M. truncatula* roots expressing *UBQ10:GFP-ABD2-GFP* was clearly disrupted after treatment with 100 nM LatB. While root epidermal cells in the elongation zone of untreated roots had extensive cortical F-actin networks, F-actin in the same region after 1 h exposure to 100 nM LatB were less dense and fragmented (**Figure 5A**). Moreover, F-actin in live *M. truncatula* roots treated with LatB had lower occupancy values, which corroborated results with chemically fixed *Z. mays* roots (**Figure 5B**). Experiments with eBL were less conclusive, particularly with regard to the impact on F-actin density. Surprisingly, F-actin in roots treated with 5 µM eBL had intact cortical F-actin networks that were qualitatively similar to untreated roots (**Figure 5A**). Quantitative analysis showed that F-actin occupancy values were consistent with the observation that 5 µM eBL had no significant effects on F-actin density in living roots (**Figure 5B**). Furthermore, while F-actin skewness increased in LatB-treated roots, this metric was not affected by 5 µM eBL (**Figure 5C**).

Next, we asked if eBL had other measurable quantitative impacts on F-actin organization in living *M. truncatula* roots. For this analysis, the degree of orientation of F-actin in root cell images was analyzed using a metric called eccentricity. Lower eccentricity values indicate a more disordered F-actin network. Modified cortical F-actin eccentricity was reported in *Physcomitrella patens* ROP4 RNA interference (RNAi) lines (Burkart et al., 2015). Using metrics developed by Burkhart et al. (2015), we found that F-actin in living *M. truncatula* roots treated with 100 nM LatB and 5 µM eBL had significantly lower eccentricity values than F-actin in untreated roots. This result indicated that both LatB and eBL treatments led to more disordered F-actin (**Figure 5D**).

### Epi-Brassinolide Inhibits Global F-Actin Dynamics in Etiolated Hypocotyls of Living *M. truncatula* Seedlings

Because actin function is defined not only by overall organization within cells, but also by dynamics (Staiger et al., 2009; Burkart et al., 2015), the effects of eBL on F-actin dynamics were analyzed. For these experiments, epidermal cells of etiolated barrel medic seedlings expressing *UBQ10:Lifeact-mGFP* were used for analyzing F-actin dynamics. We used lines expressing *UBQ10:Lifeact-mGFP* for these analyses because the F-actin arrays in hypocotyls of these lines were more readily visualized than those expressing *UBQ10:GFP-ABD2-GFP*. Roots were not used for this analysis because they grew out of the field of view within the desired 1 min period required for global actin dynamics analysis. Furthermore, the thick roots from *M. truncatula* were difficult to keep in the same focal plane during imaging, which complicated the interpretation of results.

**Figure 6:**
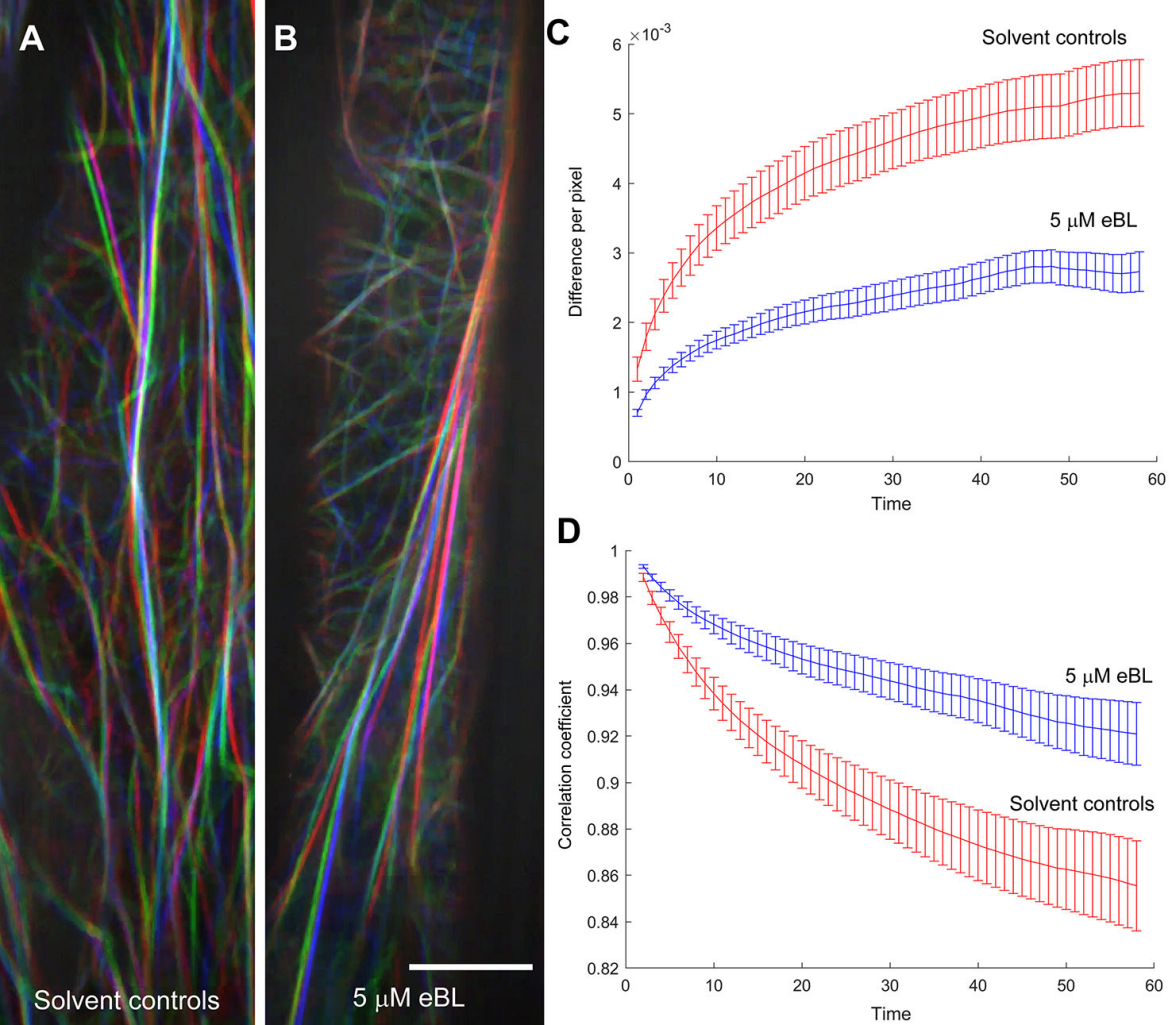
Epi-Brassinolide (eBL) inhibited global F-actin dynamics in etiolated hypocotyl epidermal cells of *M. truncatula* expressing *UBQ10:Lifeact-mGFP*. Representative images of three time points (20, 40 and 60 s) of solvent controls **(A)** and eBL treated **(B)** cells as separate color channels in red, green and blue (RGB) image. White overlay in each image indicates low dynamics. Scale bar = 10 μm. Time-lapse movies corresponding to the merged images are presented as **Supplemental Movie S1** (for untreated) and **S2** (eBL-treated). Quantification of global F-actin dynamics based on changes in pixel **(C)** and decay of the correlation coefficient **(D)**. Statistical analyses of correlation coefficients and total difference in hypocotyl epidermal cells as a function of temporal intervals. Correlation/difference for solvent controls versus eBL-treated hypocotyls are as follows: 0.02086*/0.007597* (20 s); 0.03*/0.006639* (40 s) and 0.057/0.006069* (60 s). Adjusted P-values are shown for rejecting equivalence of means; values marked by an asterisk indicate statistical significance at the 0.05 level.

Cortical F-actin in *M. truncatula* hypocotyls were captured every second for 1 min and movies were used to quantify global changes in F-actin following the metrics developed by Vidali et al. (2010) (**Supplemental Movies S1** and **S2**). These metrics included pixel difference values that capture intensity differences between images every second, and correlation coefficients that indicate the degree of change (**Figures 6A, B**). A larger difference between pixels over time and a steeper decay in the correlation coefficient indicated a more dynamic F-actin network (Vidali et al., 2010; Dyachok et al., 2014; Burkart et al., 2015). The F-actin network in epidermal cells of eBL-treated etiolated *M. truncatula* hypocotyls had lower intensity difference values and a more gradual decay of the correlation coefficient compared to solvent control seedlings (**Figures 6C, D**). Taken together, these data imply that eBL treatment suppressed F-actin dynamics in etiolated *M. truncatula* hypocotyls.

## Discussion

In this paper, a slowly-rotating 2-D clinostat was used to gain additional insights into actin-mediated regulation of directional root growth. These experiments showed that brassinosteroids were involved in modulating directional root growth, in part, by modifying the organization and dynamics of the actin cytoskeleton. This conclusion was based on the observation that exogenous application of eBL and LatB triggered similar enhanced root gravitropism and amplified root coiling on a clinostat (**Figure 1**). The curvature responses of roots treated with eBL and LatB during continuous gravistimulation and on a clinostat are reminiscent of the behavior of inflorescence stems of *A. thaliana myosin XI* and *actin8* mutants (Kato et al., 2010; Okamoto et al., 2015; Talts et al., 2016). The hyperbending of inflorescence stems of myosin and actin *A. thaliana* mutants was proposed to be the result of inhibited autotropism. With an aberrant acto-myosin system in fiber cells, it was proposed that the stems could no longer perceive their posture to initiate a straightening response (Okamoto et al., 2015). Here, we propose that a similar mechanism may be operating in roots, and that eBL is a component of the root organ straightening response through its impacts on F-actin.

Most of the studies implicating the actin cytoskeleton in gravitropism have emphasized its role in the gravity perception phase (Blancaflor, 2013; Su et al., 2017). In gravity sensing cells such as the columella, actin has been proposed to interact with amyloplasts to facilitate conversion of the physical gravity signal to a biochemical signal (Zheng et al., 2015). Because actin inhibitors such as LatB or mutations to genes encoding actin itself led to stronger root gravitropic curvature (Yamamoto and Kiss, 2002; Hou et al., 2003; Kato et al., 2010; Lanza et al., 2012), it was proposed that the unrestrained sedimentation of amyloplasts may amplify the gravity sensing signal leading to a more robust graviresponse (Blancaflor, 2013). However, work on *A. thaliana myosin XI* mutants (Okamoto et al., 2015) and results presented here, suggest a more prominent role for actin in the graviresponse phase, and more specifically in the autotropic straightening response that enables plant organs to correct their orientation (Stankovic et al., 1998a; Stanković et al., 1998b). Furthermore, given the difficulties in obtaining clear images of F-actin in the columella (**Figure 3**), we cannot rule out the possibility that a secondary mechanism of gravity sensing in the root transition zone might be affected by LatB (Wolverton et al., 2002).

A number of studies have implicated eBL in directional root growth control. For instance, eBL application mirrored the enhanced root waving and gravitropism of *A. thaliana actin2* mutants and caused disruptions in F-actin configurations in wild type (Lanza et al., 2012). Enhanced root gravitropism was also observed in wild-type *A. thaliana* roots treated with eBL and this process was proposed to be mediated by ROP2-dependent modification of F-actin assembly and disassembly (Li et al., 2005). While our results expand on these previous observations, experiments with a clinostat implicated eBL as a potential regulator of autotropism, and like previous results with LatB-treated roots, provide an alternative explanation of why this hormone has an apparent promotive effect on gravitropism. Although previous studies have shown that eBL modifies F-actin organization in *A. thaliana* roots (Lanza et al., 2012), we found that eBL effects on F-actin organization in fixed *Z. mays* roots and living *M. truncatula* roots were not as pronounced and consistent as those induced by LatB. Higher concentrations of eBL were needed to elicit distinct qualitative and quantitative changes in F-actin in both chemically-fixed *Z. mays* roots and living *M. truncatula* roots. In fact, it was surprising to find that 1 µM eBL led to increased F-actin occupancy values compared to controls, while concentrations lower (500 nM) or higher (5 µM) than 1 µM led to reduced values (**Figure 2B**). Moreover, F-actin occupancy and bundling in living roots treated with 5 µM eBL was not different from untreated controls (**Figures 5B, C**). LatB-treatment on the other hand, led to an increase in F-actin bundling, which was likely due to aggregation of F-actin fragments in some of the root cells analyzed. Although occupancy and bundling were not affected by eBL, we found that eccentricity of F-actin in LatB- and eBL-treated roots were reduced relative to controls (**Figure 5D**). It is tempting to speculate that eBL-induced reduction in F-actin alignment alone is sufficient to account for a large part of the root autotropic inhibition observed here. It is important to note, however, that all of the F-actin analyses reported here were conducted pre-clinorotation. It is possible that changes in F-actin organization during the process of clinorotation were unaccounted for in our analyses. Experiments involving chemically fixing roots at various time points during clinorotation or a system in which live roots expressing the F-actin reporters can be imaged on a clinostat, will be necessary to address such possibilities.

Okamoto et al. (2015) proposed that the long F-actin cables in the inflorescence stem fiber cells were the structures that modulated organ straightening possibly by regulating cytoplasmic streaming. It is possible that eBL regulation of root autotropic straightening is facilitated by a population of F-actin in specific root cell types that are not detected by the quantitative metrics used in our studies. This could explain why eBL had only modest effects on overall F-actin organization, but still caused LatB-like autotropic straightening defects. Another possibility is that dampening of global F-actin dynamics by eBL as revealed by live cell imaging of etiolated *M truncatula* hypocotyls could account for autotropic straightening defects. Suppressed global F-actin dynamics after eBL exposure could modify targeting of plasma membrane-associated proteins, such as PIN2, leading to dampened autotropism (Li et al., 2005; Lanza et al., 2012). Unfortunately, we were unable to obtain global F-actin dynamic data on *M. truncatula* root cells because of technical difficulties associated with keeping actively growing roots within the microscope field of view. As such, a direct link between root F-actin dynamics and root autotropism is missing. For the future, a suitable clinostat assay using *M. truncatula* shoots should help clarify if reduced global F-actin dynamics can explain eBL effects on organ straightening. Furthermore, live fluorescent protein-based F-actin marker lines in *Z. mays* could help strengthen the link between F-actin organization and autotropic organ growth (Wu et al., 2013).

For more than a decade, genetically-encoded F-actin reporters based on fluorescent protein technology have advanced our understanding of actin function during plant development (Voigt et al., 2005; Wang et al., 2008). In addition to new insights into the processes underlying directional root growth control, our work introduces a set of *M. truncatula* lines expressing two types of genetically encoded F-actin reporters. These new F-actin reporter lines could potentially be used to address a range of biological questions. Specifically, a future gravitational biology-related study in which these new *M. truncatula* lines would be of value is the dynamic monitoring of F-actin in various tissues of graviresponding roots using vertical stage microscopy (Von Wangenheim et al., 2017). Vertical stage microscopy combined with new imaging modalities such as airy scan should enable super-resolution of F-actin during plant gravity response (Komis et al., 2018). Moreover, some light sheet microscopy platforms allow for plant organs to be positioned vertically. Such methods pave the way for long term and volumetric imaging of F-actin dynamics during plant gravity response using these new *M. truncatula* lines (Ovečka et al., 2018). Similar to previous reports in *A. thaliana* plants expressing live cell F-actin reporters (Ketelaar et al., 2004; Dyachok et al., 2014), mature *M. truncatula* plants expressing *Lifeact-mGFP* and *GFP-ABD2-GFP* appear to have growth defects based on their reduced shoot and root dry weights when compared to controls. However, the negative growth effects induced by expressing these reporters were mild and not very pronounced in young seedlings. Thus, if used with caution, these Lifeact-mGFP and GFP-ABD2-GFP *M. truncatula* lines should be a valuable addition to existing fluorescent protein-based toolkit for studying dynamic cellular processes in legumes (Ivanov and Harrison, 2014; Cvrckova and Oulehlova, 2017; Zhang et al., 2019).

In conclusion, our results implicate brassinosteroids in root autotropism. Brassinosteroids modulate this process by modifying F-actin organization and dynamics in a manner different from that of the actin-disrupting compound LatB. Regardless of the mechanisms by which eBL modifies F-actin, our studies reinforce previous observations that the acto-myosin system is an important component of plant tropistic responses. Finally, through our work, we introduced a set of *M. truncatula* lines expressing fluorescent protein-based reporters that could enable new studies of actin-mediated processes in legumes.

## Data Availability Statement

All datasets for this study are included in the article/**Supplementary Material**.

## Author Contributions

LB, AP-G, AC, and EB conceived and designed the research. LB, JK, and EB conducted F-actin labelling in fixed roots. LB, AP-G, SC, AC, and EB performed root gravitropism, plant growth, clinostat assays and live cell imaging. QJ and JS generated and maintained transgenic plants. FL conducted quantitative analysis of F-actin. All authors contributed to writing and editing the paper.

## Funding

This work was supported by the National Aeronautics and Space Administration grants 80NSSC18K1462 and 80NSSC19KO129 to EB, and the Noble Research Institute LLC. LB was supported by a fellowship from Biomass for the 21st century (https://b21st.ku.dk/).

## Conflict of Interest

The authors declare that the research was conducted in the absence of any commercial or financial relationships that could be construed as a potential conflict of interest.
