# Supplementary material for: Brassinosteroids Inhibit Autotropic Root Straightening by Modifying Filamentous-Actin Organization and Dynamics
Source: Front Plant Sci. 2020 Feb 4;11:5. doi: 10.3389/fpls.2020.00005 (PMC7010715; doi:10.3389/fpls.2020.00005)
Supplement: Supplementary file 1 [file DataSheet_1.pdf]

Appendix 1

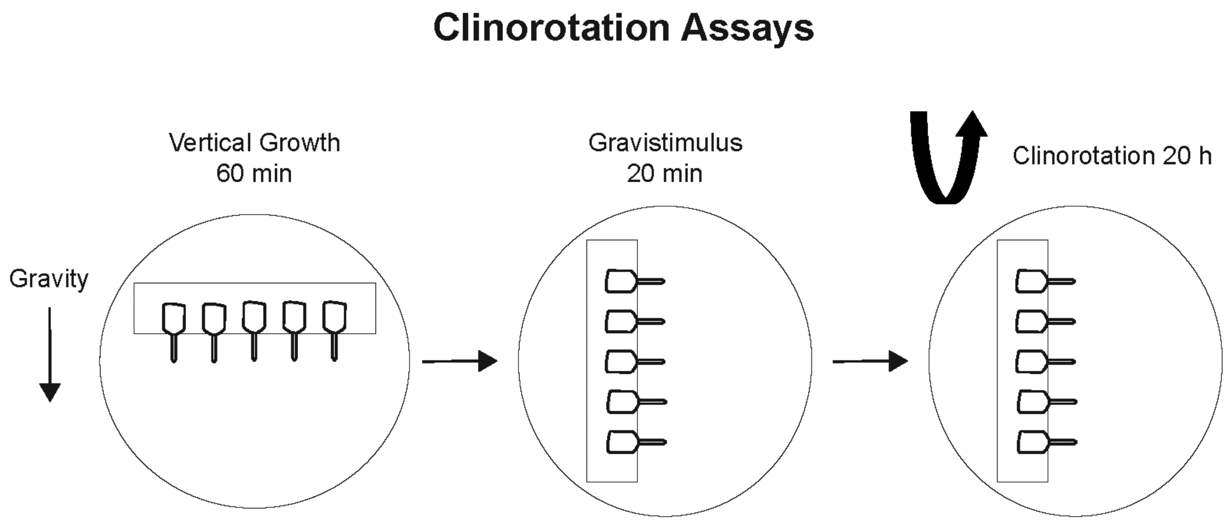

Appendix 1. Schematic depicting clinorotation assays used for *Z. mays* roots.
