# Supplementary material for: Brassinosteroids Inhibit Autotropic Root Straightening by Modifying Filamentous-Actin Organization and Dynamics
Source: Front Plant Sci. 2020 Feb 4;11:5. doi: 10.3389/fpls.2020.00005 (PMC7010715; doi:10.3389/fpls.2020.00005)
Supplement: Supplementary file 2 [file DataSheet_2.pdf]

Supplementary Material

Supplementary Movie Legends

Supplementary Movie 1. Spinning-disc confocal microscopy of F-actin dynamics in etiolated hypocotyl epidermal cells treated with the solvent control solution. Each frame was taken every 1 second and the total elapsed time is 1 minute.

Supplementary Movie 2. Spinning-disc confocal microscopy of F-actin dynamics in etiolated hypocotyl epidermal cells treated with 5 μM eBL. Each frame was taken every 1 second and the total elapsed time is 1 minute.
